# Supplementary material for: Strigo-D2—a bio-sensor for monitoring spatio-temporal strigolactone signaling patterns in intact plants
Source: Plant Physiol. 2021 Oct 29;188(1):97–110. doi: 10.1093/plphys/kiab504 (PMC8774841; doi:10.1093/plphys/kiab504)
Supplement: kiab504_Supplementary_Data [file kiab504_supplementary_data.zip › kiab504-suppl_data/PP2021BT01092R1 Supplemental Movie Legends.docx]

**Supplemental Movie S1** **Strigo-D2 response to (+)-5DS treatment in cotyledons.** Shown are overlays of mVenus and mCherry-derived signals. Green: mVenus. Magenta: mCherry. Time after (+)-5DS treatment is indicated at the upper left corner of the movie. Scale bar: 50 μm. Same settings were applied in Movies 2, 3, 4, and 6.

**Supplemental Movie S2** **Strigo-D2 response to (+)-5DS treatment in hypocotyls.**

**Supplemental Movie S3**  **Strigo-D2 response to (+)-5DS treatment in root maturation zones.**

**Supplemental Movie S4**  **Strigo-D2 response to (+)-5DS treatment in root tips.**

**Supplemental Movie S5** **Gradual decrease of Strigo-D2 responsiveness to (+)-5DS from the maturation zone to the very tip of the root.** Shown are overlays of mVenus, mCherry, and brightfield-derived signals. Green: mVenus. Magenta: mCherry. Grey: brightfield. Time after (+)-5DS treatment is indicated at the upper left corner of the movie. Scale bar: 50 μm.

**Supplemental Movie S6**  **Comparison between Strigo-D2 response to (+)-5DS in pavement and in guard cells.**
