# Supplementary material for: Strigo-D2—a bio-sensor for monitoring spatio-temporal strigolactone signaling patterns in intact plants
Source: Plant Physiol. 2021 Oct 29;188(1):97–110. doi: 10.1093/plphys/kiab504 (PMC8774841; doi:10.1093/plphys/kiab504)
Supplement: kiab504_Supplementary_Data [file kiab504_supplementary_data.zip › kiab504-suppl_data/Supplemental Data.pdf]

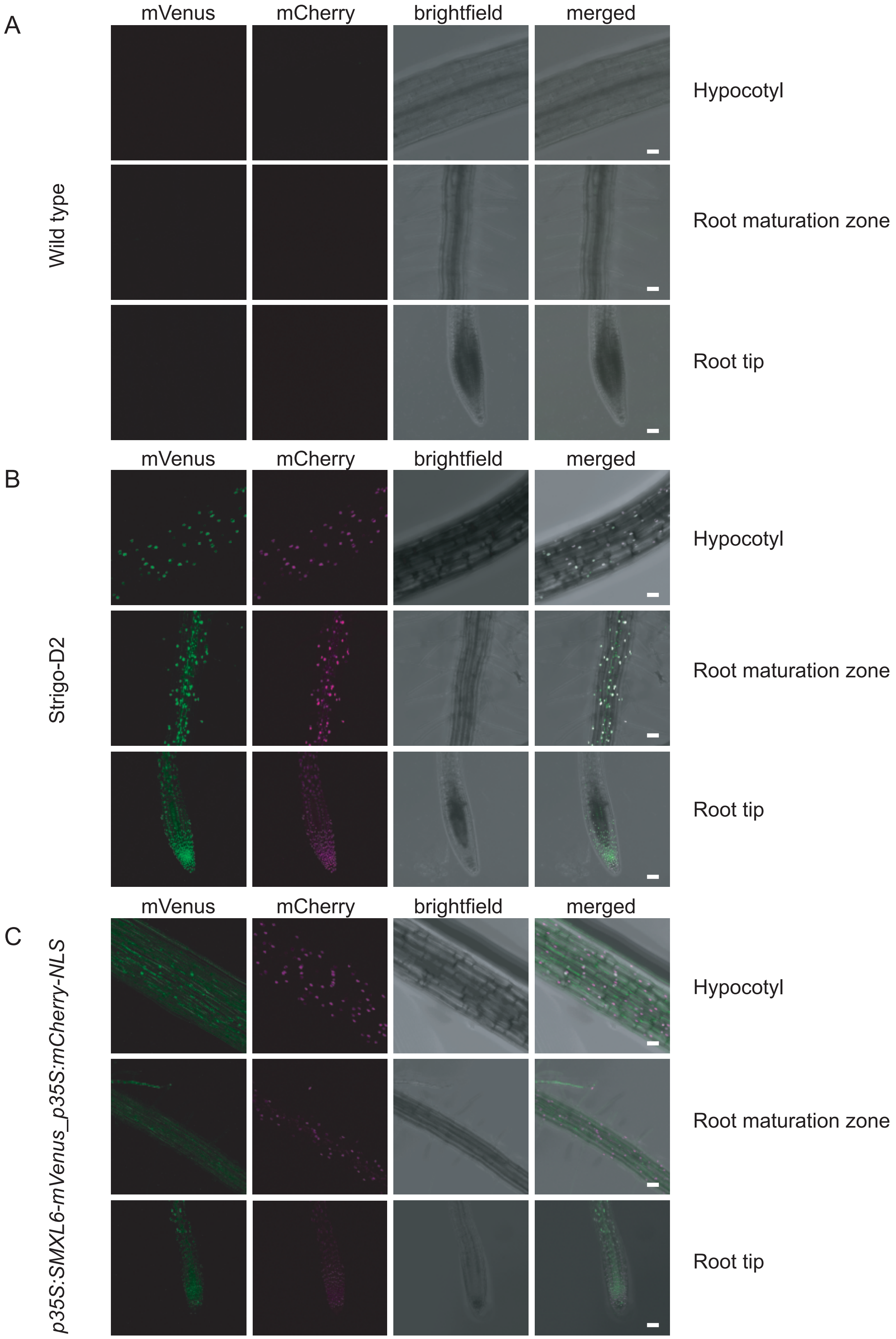

Supplemental Figure S1. Comparison of fluorescent signals in transgenic seedlings carrying Strigo-D2 or *p35S:SMXL6-mVenus_p35S:mCherry-NLS* transgenes. (A) Wild type seedling without transgene. Laser excitation intensity for mVenus: 1 %, Detection wavelength range 519-555 nm. (B) Strigo-D2 seedling. Laser excitation intensity for mVenus: 1 %, Detection wavelength range 524-540. (C) *p35S:SMXL6-mVenus_p35S:mCherry-NLS* seedling. Laser excitation intensity for mVenus: 10 %, Detection wavelength range 519-555. Scale bars: 50 μm.

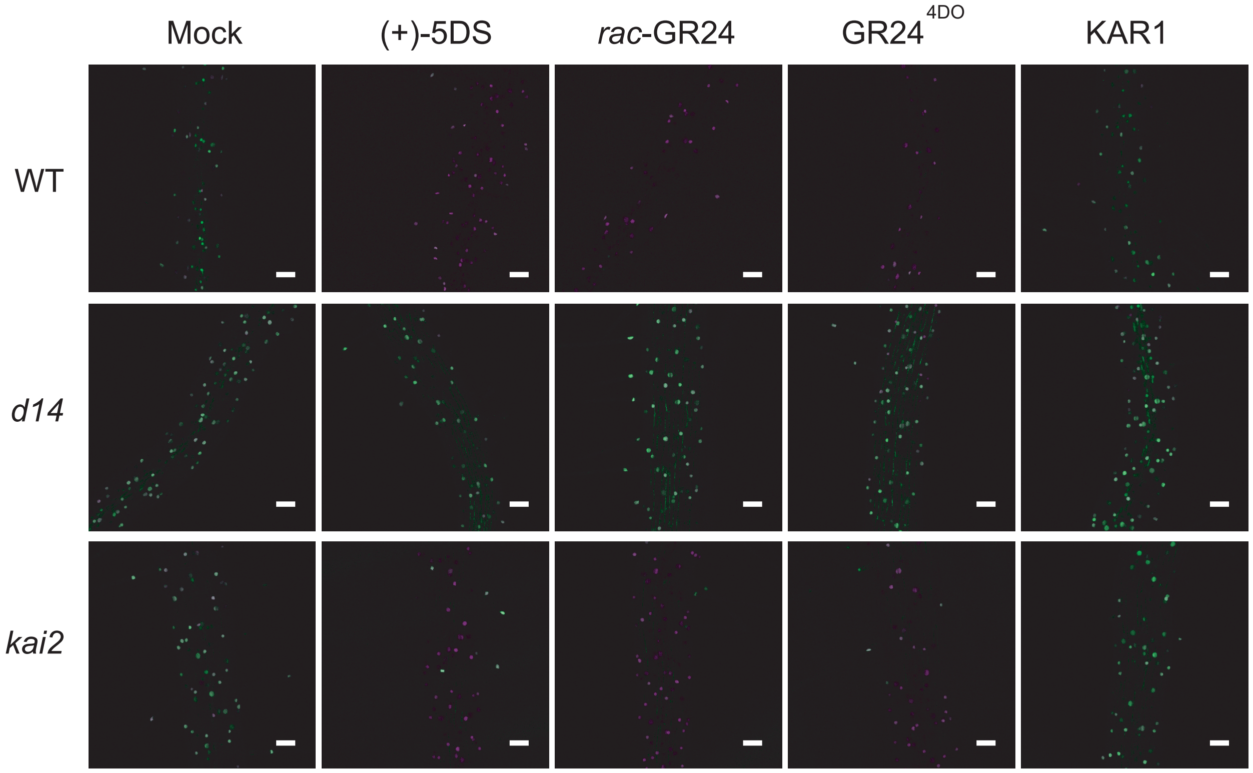

Supplemental Figure S2. Effect of SLs and KAR1 on Strigo-D2 in the root maturation zone of different genetic backgrounds. Shown are overlays of mVenus and mCherry-derived signals. Green: mVenus. Magenta: mCherry. Treatment duration was 120 min. Scale bars: 50 µm.

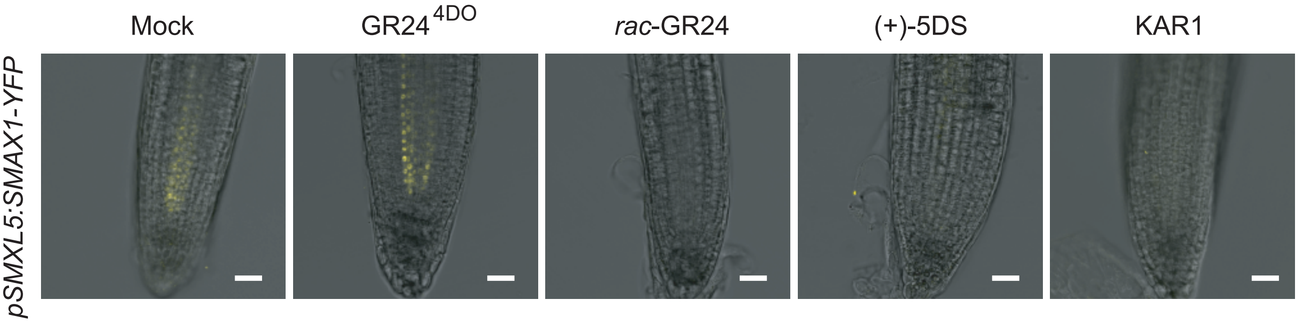

Supplemental Figure S3. Effect of SLs and KAR1 on *pSMXL5:SMAX1-YFP* reporter activity. YFP-derived signal is visualized in yellow. Treatment duration was 30 min. Scale bars: 50 µm.

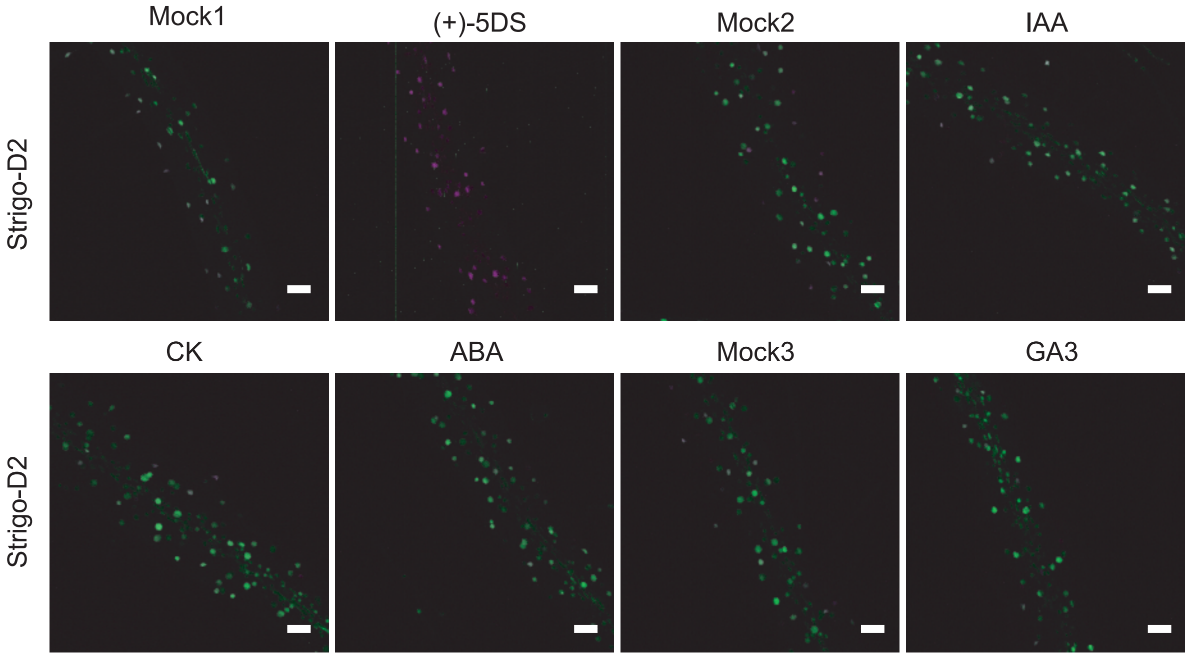

Supplemental Figure S4. Strigo-D2 response to (+)-5DS and other plant hormones in the root maturation zone. Images merging mCherry (magenta) and mVenus (green)-derived signals are shown. Treatment duration was 120 min. Mock1: treatment with (+)-5DS solvent. Mock2: treatment with IAA, CK, and ABA solvent. Mock3: treatment with GA_3 solvent. IAA, 3-Indoleacetic acid. CK, Cytokinin (*trans*-Zeatin). ABA, (+/-)-Abscisic acid. GA_3, Gibberellic acid. Scale bars: 50 μm.

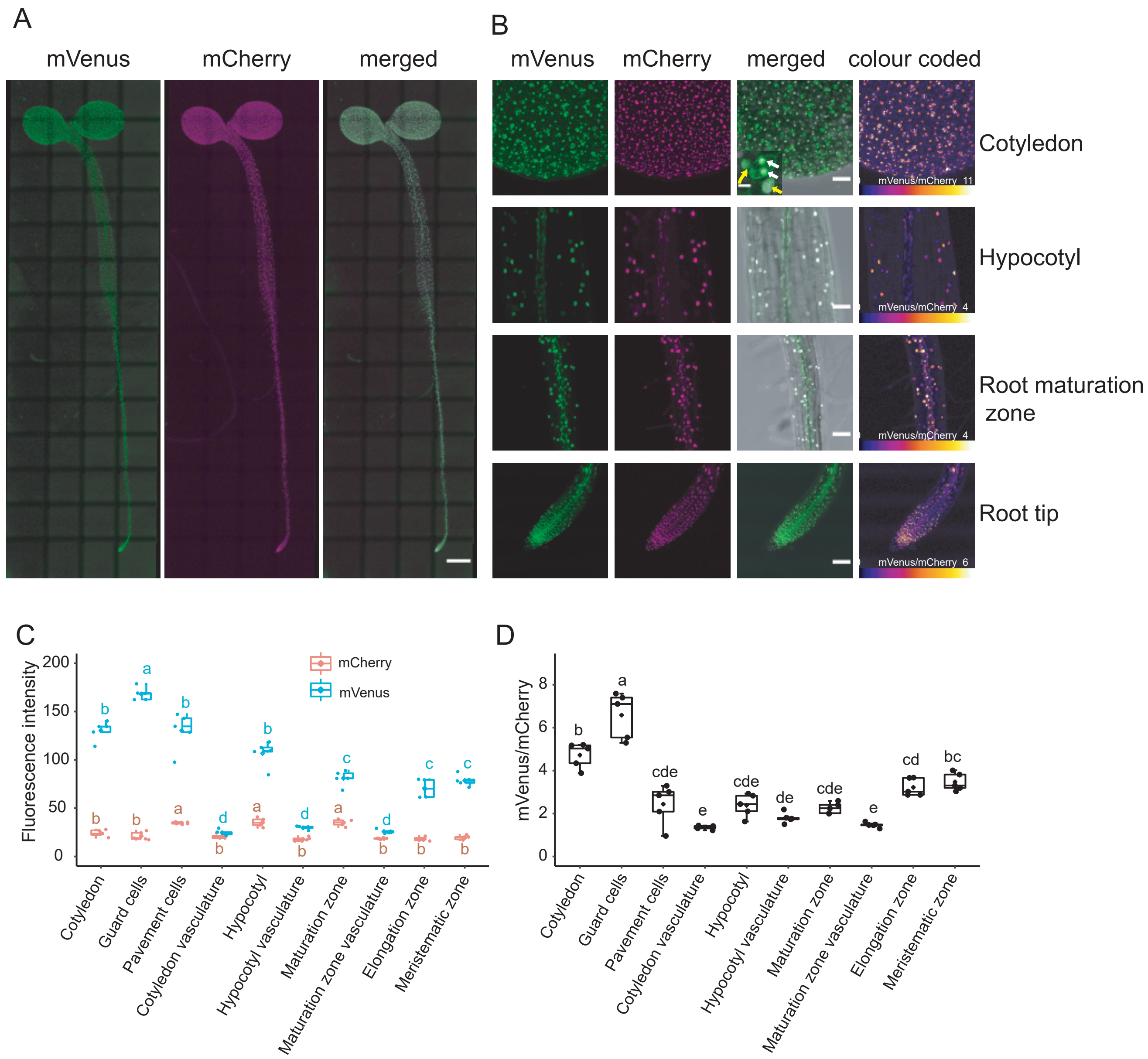

Supplemental Figure S5. Strigo-D2 activity in seedlings. (A) Ubiquitous expression of Strigo-D2 in seedlings. Scale bar: 500 µm. (B) Activity of Strigo-D2 in cotyledons, hypocotyls, root maturation zones and root tips. Images were recaptured from corresponding positions in (A) with higher magnification. Scale bars: 50 µm. White arrows: guard cells. Yellow arrows: pavement cells. Scale bar in magnified image: 10 µm. The merged and colour coded images in (A) and (B) are the same as those in Figure 4A-I. (C) Fluorescence intensity of mVenus and mCherry detected in different tissues or cells. (D) Intensity ratios of mVenus and mCherry in different tissues or cells. Different letters above boxes indicate statistical groups (One-way ANOVA, post hoc LSD test with Bonferroni adjustment, $p < 0.01$, n = 5).

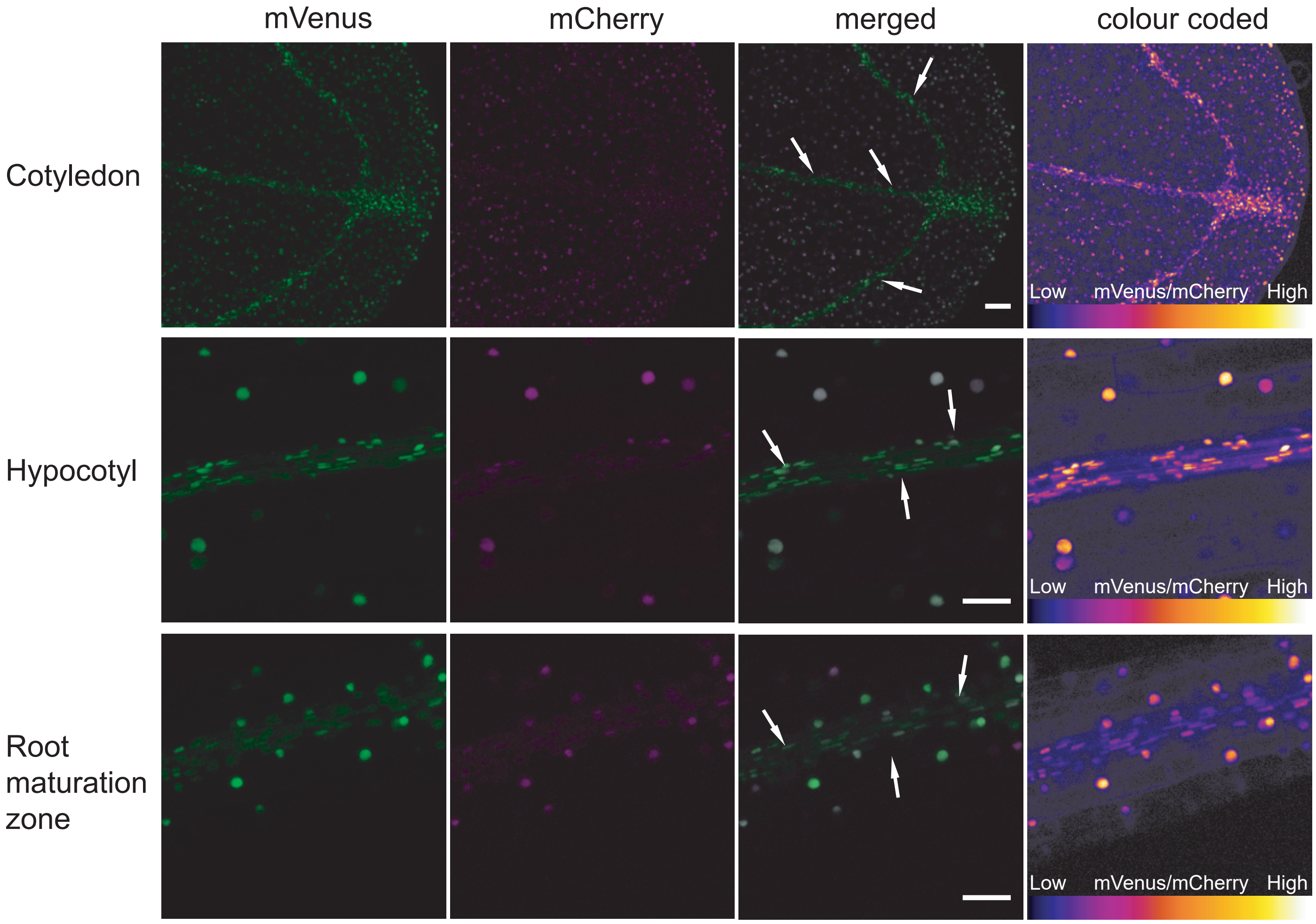

Supplemental Figure S6. Strigo-D2 activity in the vasculature of cotyledons, hypocotyls and root maturation zones. White arrows indicate vascular strands. Scale bars: 50 µm.

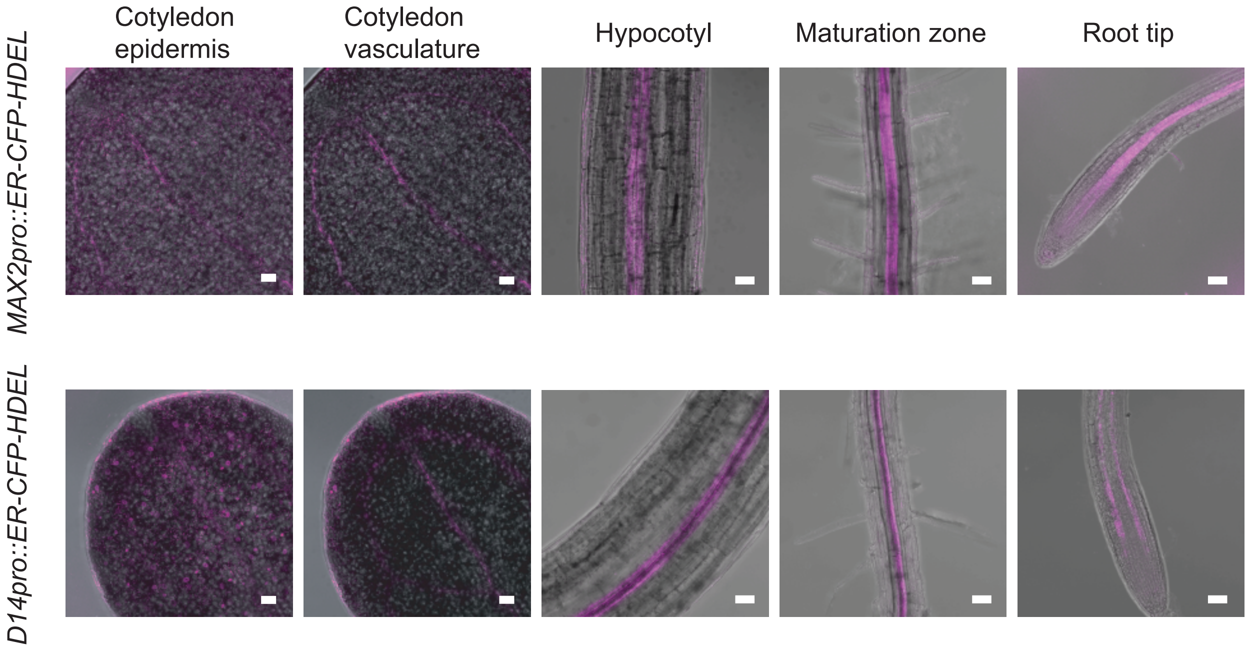

Supplemental Figure S7. *D14* and *MAX2* promoter reporter activity in seedlings. CFP fluorescence is visualized in magenta. Scale bar: 50 µm.

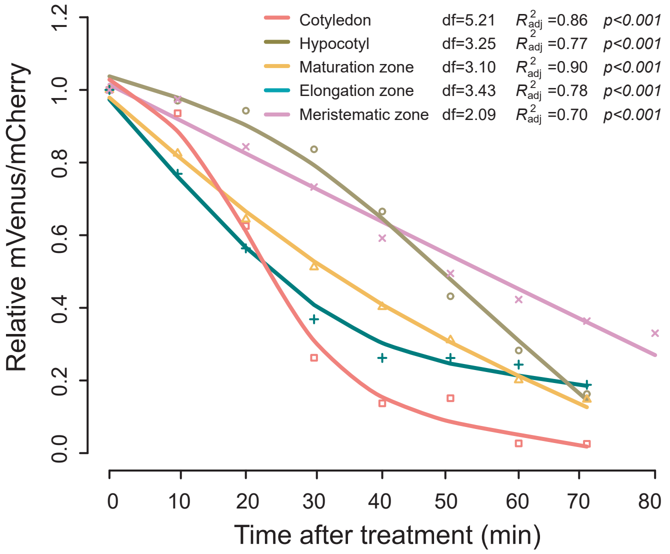

Supplemental Figure S8. Spline regression of relative mVenus/mCherry as function of treatment time in different tissues. Curves of best fit were computed in R using ss Function in the npreg Package. Curves in were generated by non-linear regression and computed in R using the ss Function in the npreg Package. df: degree of freedom. Data points are mean values of five biological replicates. Adjusted R squares indicate how well generated curves fit obtained data points. *p*-values for the F-test indicate significance levels of the correlation between the models and obtained data.

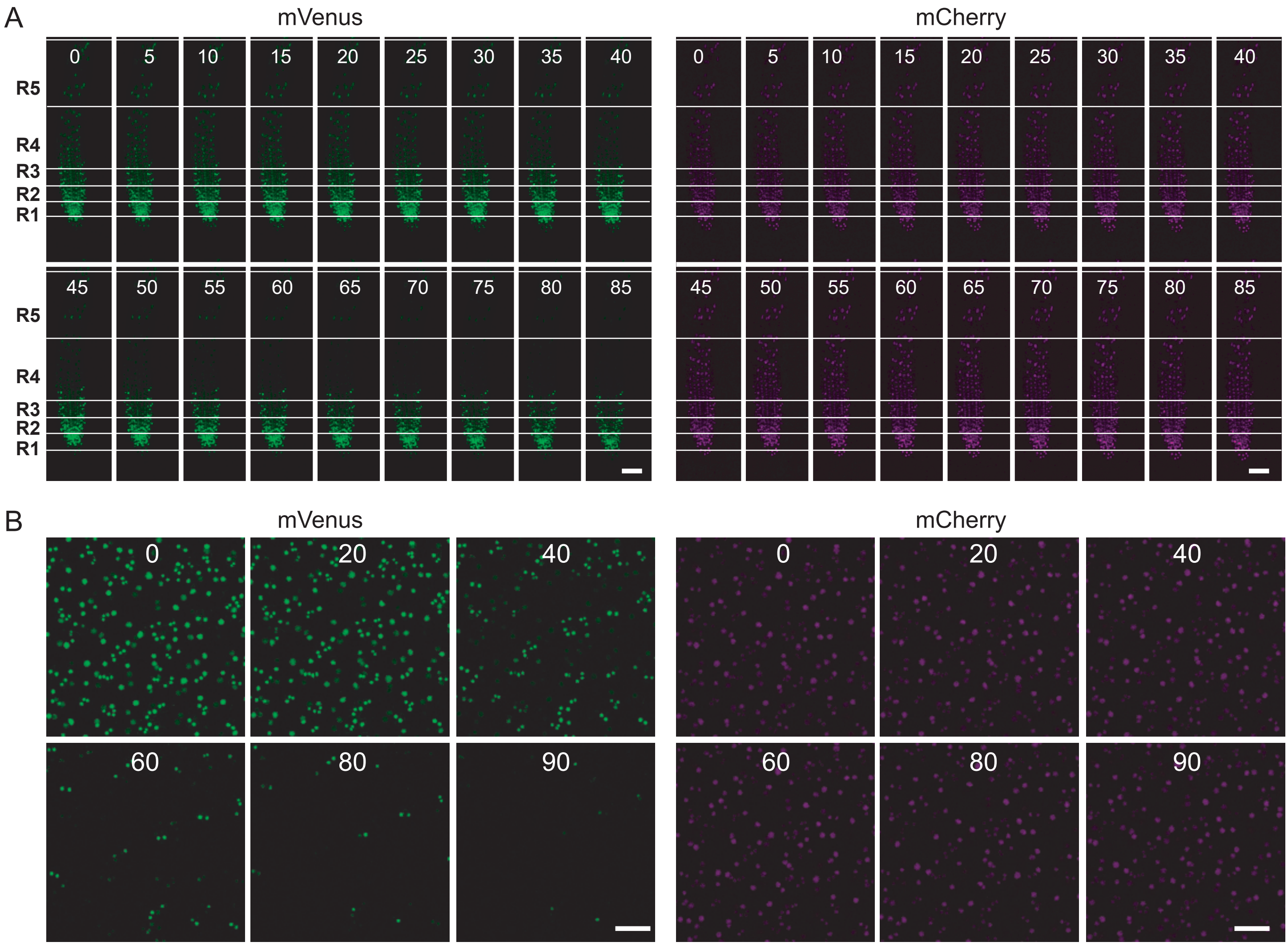

Supplemental Figure S9. Dual-channel representation of Strigo-D2 response to (+)-5DS at a cellular resolution in root tips and cotyledon epidermis. (A) Confocal images of root tips at the indicated time points (in minutes, shown above each image) after application. Concentration of (+)-5DS: 0.5 μM. Covered range of the regions (distance from bottom of meristematic zone): R1: 0-65 μm, R2: 65-130 μm, R3: 130-195 μm, R4: 195-395 μm, R5: 395-595 μm. (B) Confocal images of the cotyledon epidermis at the indicated time points (in minutes, shown above each image) after application. The images in (A) and (B) are the same as those in Figure 6 (A and C). Scale bars: 50 μm.

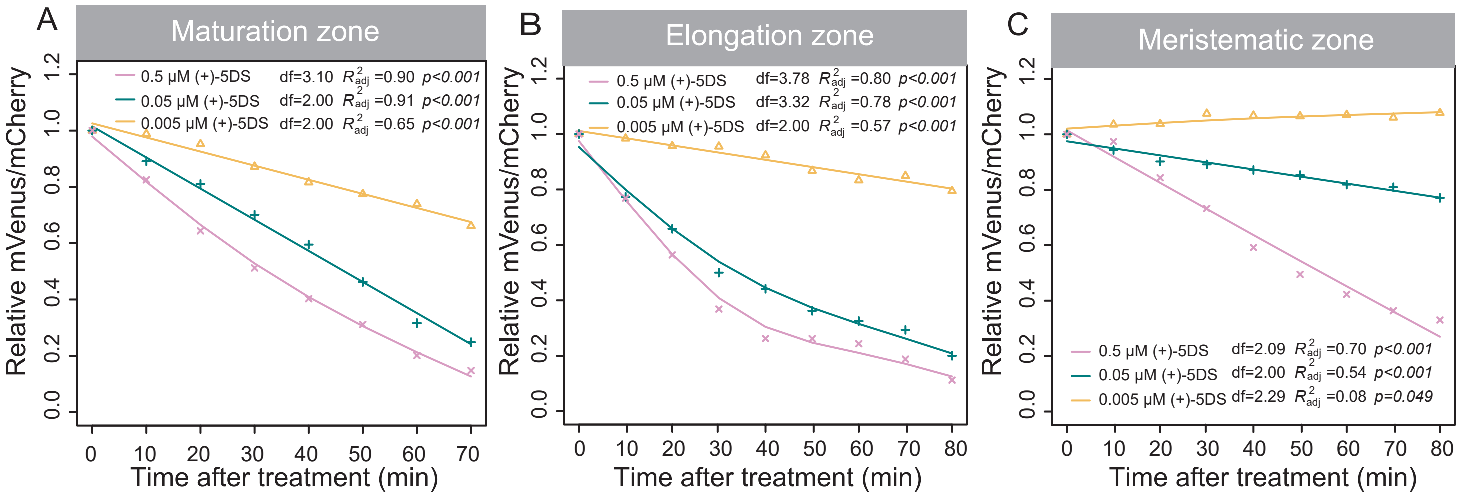

Supplemental Figure S10 Spline regression of relative mVenus/mCherry as function of treatment time under gradient (+)-5DS concentrations in different zones of the root. (A) maturation zone. (B) elongation zone. (C) meristematic zone. Curves in were generated by non-linear regression and computed in R using the ss Function in the npreg Package. df: degree of freedom. Data points are mean values of five biological replicates. Adjusted R squares indicate how well generated curves fit obtained data points. *p*-values for the F-test indicate significance levels of the correlation between the models and obtained data.

Supplemental Table S1 GreenGate Vectors used in this study

Vector name	Vector ID	Modules
p35S:SMXL6-mVenus_p35S:mCherry-NLS	*pCS12*	*35S* (*pGGA004**), B-dummy (*pGGA022**), *pGGC-SMXL6* (*pCS1*), *pGGD-mVENUS* (*pD00587* ‡), *rbscT* (*pGGE001**), F-H adapter (*pGGG001**), H-A adapter (*pGGG002**), *mCherry* (*pGGC015**), *NLS* (*pGGD007**), *BastaR* (*pGGF001**), Destination vector (*pGGZ003**)
p35S:SMXL6-D2-mVenus_p35S:mCherry-NLS	*pJZ27*	*35S* (*pGGA004**), B-dummy (*pGGA022**), *pGGC-SMXL6-D2* (*pJZ25*), *pGGD-mVENUS* (*pD00587* ‡), *rbscT* (*pGGE001**), F-H adapter (*pGGG001**), H-A adapter (*pGGG002**), *mCherry* (*pGGC015**), NLS (*pGGD007**), *BastaR* (*pGGF001**), Destination vector (*pGGZ003**)
D14pro:ER-CFP_WOXpro:ER-YFP	*pVJ33*	*D14pro* (*pVJ25*), ER Signal Peptide (*pGGB006**), *mTurquoise2* (*pSW596*†), HDEL (*pGGD008**), *ter AT4G24550* (*pVL12*†), F-H adapter (*pGGG001**), H-A adapter (*pGGG002**), *WOX4pro* (*pVL37*†), *mVenus* (*pSW549*†), *tWOX4* (*pVL22*†), *BastaR* (*pGGF001**), Destination vector (*pGGZ003**)
MAX2pro:ER-CFP_ WOXpro:ER-YFP	*pVJ47*	*MAX2pro* (*pVJ40*), ER Signal Peptide (*pGGB006**), *mTurquoise2* (*pSW596*†), HDEL (*pGGD008**), *ter AT4G24550* (*pVL12*†), F-H adapter (*pGGG001**), H-A adapter (*pGGG002**), *WOX4pro* (*pVL37*†), mVenus (*pSW549*†), *tWOX4* (*pVL22*†), *BastaR*, (*pGGF001**), Destination vector (*pGGZ003**)

*reference: (Lampropoulos et al., 2013)

†reference: (Schürholz et al., 2018)

‡reference: (Wallner et al., 2019)

Supplemental Table S2 Primers used in this study

Primer name	Sequence (5'-3')	Usage
SMXL6-1st-F	AACAGGTCTCAGGCTATGCCGAC GCCGGTGACTACGG	Cloning 1st part of *SMXL6* into *pGGC000*
SMXL6-1st-R	AACAGGTCTCTCGCCTGTGACCG TTTGATCGCCGC	Cloning 1st part of *SMXL6* into *pGGC000*
SMXL6-2nd-F	AAACGGTCTCAGGCGAACCAGAG	Cloning 2nd part of *SMXL6* into *pGGC000*
SMXL6-2nd-R	TTCTTTGGTCTCAATAGCCCGTAG CC	Cloning 2nd part of *SMXL6* into *pGGC000*
SMXL6-3rd-F	AACAGGTCTCGCTATTGAAACCAA AGAAGACAAGGGAATAACAGGC	Cloning 3rd part of *SMXL6* into *pGGC000*
SMXL6-3rd-R	AACAGGTCTCACTGACCATATCAC ATCCACCTTCGCC	Cloning 3rd part of *SMXL6* into *pGGC000* and *SMXL6-D2* cloning
E2-5-bp-deletion-F	GCTTTAGATGACGCTAATACATCA GCC	In fusion of *SMXL6* parts
E2-5-bp-deletion-R	CTTACTGCTGCCTGTTATTCCCTT G	In fusion of *SMXL6* parts
SMXL6-frag1-into-E2-F	TGAAGCTTGGTCTCAGGCTATGC C	In fusion of *SMXL6* parts
SMXL6-frag1-into-E2-R	CGTCTCTGGTTCGCCTGTGACCG TTTGATCGCCG	In fusion of *SMXL6* parts
SMXL6-D2-F	AACAGGTCTCAGGCTCAATGCAG AAAGATTTCAAGTCTC	*SMXL6-D2* cloning
pMAX2_ModuleA F	AACAGGTCTCAACCTTGGAACGA ACGTGGAGATCG	*MAX2* promoter cloning
pMAX2_ModuleA R	AACAGGTCTCATGTTGAGAAGCG GCAAATCTACAA	*MAX2* promoter cloning
pD14_ModuleA F	AACAGGTCTCAACCTAAATGTCTT AACCATCTTAA	*D14* promoter cloning
pD14_ModuleA R	AACAGGTCTCATGTTTTTTTTATGT GTTTGGGTTTG	*D14* promoter cloning
